# BDNF as a biomarker for neuropathic pain: Consideration of mechanisms of action and associated measurement challenges

**DOI:** 10.1002/brb3.2903

**Published:** 2023-02-01

**Authors:** Bhushan Thakkar, Edmund O. Acevedo

**Affiliations:** ^1^ Department of Physical Therapy Virginia Commonwealth University Richmond Virginia USA; ^2^ Department of Kinesiology and Health Sciences Virginia Commonwealth University Richmond Virginia USA

**Keywords:** BDNF, biomarker, measurement, mechanisms, neuropathic pain

## Abstract

**Introduction:**

The primary objective of this paper is to (1) provide a summary of human studies that have used brain derived neurotrophic factor (BDNF) as a biomarker, (2) review animal studies that help to elucidate the mechanistic involvement of BDNF in the development and maintenance of neuropathic pain (NP), and (3) provide a critique of the existing measurement techniques to highlight the limitations of the methods utilized to quantify BDNF in different biofluids in the blood (i.e., serum and plasma) with the intention of presenting a case for the most reliable and valid technique. Lastly, this review also explores potential moderators that can influence the measurement of BDNF and provides recommendations to standardize its quantification to reduce the inconsistencies across studies.

**Methods:**

In this manuscript we examined the literature on BDNF, focusing on its role as a biomarker, its mechanism of action in NP, and critically analyzed its measurement in serum and plasma to identify factors that contribute to the discrepancy in results between plasma and serum BDNF values.

**Results:**

A large heterogenous literature was reviewed that detailed BDNF's utility as a potential biomarker in healthy volunteers, patients with chronic pain, and patients with neuropsychiatric disorders but demonstrated inconsistent findings. The literature provides insight into the mechanism of action of BDNF at different levels of the central nervous system using animal studies. We identified multiple factors that influence the measurement of BDNF in serum and plasma and based on current evidence, we recommend assessing serum BDNF levels to quantify peripheral BDNF as they are more stable and sensitive to changes than plasma BDNF.

**Conclusion:**

Although mechanistic studies clearly explain the role of BDNF, results from human studies are inconsistent. More studies are needed to evaluate the methodological challenges in using serum BDNF as a biomarker in NP.

## Graphical Abstract

Preclinical studies support the role of BDNF as a biomarker in Neuropathic Pain. This study identified several methodological considerations that need to be accounted for when measuring BDNF peripherally. Additional longitudinal investigations are needed to develop standardized guidelines to measure serum BDNF and to clearly determine its role in the diagnosis and treatment of neuropathic pain.

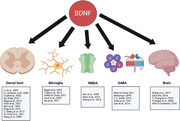

1

The International Association for the Study of Pain defines neuropathic pain (NP) as “pain caused by a lesion or disease of the somatosensory nervous system” (Loeser & Treede, 2008). It can be initiated by nerve, brain, or spinal cord injury and represents a broad category of pain syndromes encompassing a wide variety of peripheral of central disorders. Previous epidemiological studies have revealed that NP affects 7−10% of the general population (Bouhassira et al., 2008; Colloca et al., 2017; Torrance et al., 2006; van Hecke et al., 2014), accounting for almost 20–25% of patients with chronic pain (Bouhassira et al., 2008; Dahlhamer et al., 2018). It is more frequent in older individuals (> 60 years old), more common in women than in men and characterized by unpleasant symptoms, such as shooting or burning pain, numbness, and allodynia (Bouhassira, 2019; Bouhassira et al., 2008; Torrance et al., 2006). It is also associated with a high level of disability (Attal et al., 2011; Doth et al., 2010; Gálvez et al., 2007) and has a high socioeconomic cost (Attal et al., 2011; Jensen et al., 2007; B. H. Smith et al., 2007; van Hecke et al., 2014). Most importantly, current drug treatment is inadequate due to both poor efficacy and tolerability (Bannister et al., 2020; Cavalli et al., 2019; Taneja et al., 2017). A recent report by Maher and colleagues using clinical trial data from the last 20 years reported that the probability of successful drug treatment for NP was only 7.1% (Maher et al., 2022). Identifying effective treatments to address the associated severe pain and disability is limited by the lack of understanding of the underlying pathophysiological mechanisms (Cavalli et al., 2019; Finnerup et al., 2020; Price & Gold, 2018; Szok et al., 2019). The identification of a biomarker that links the signs and symptoms of NP to pathophysiological mechanisms, would provide decisive information relevant to drug‐discovery and development. This review examines the literature on brain derived neurotrophic factor (BDNF) to determine the physiological validity for utilizing BDNF as a biomarker, and the possibility of a pragmatic approach to measuring BDNF peripherally in the blood.

The subjective self‐reporting of pain has played a key role in the diagnosis and treatment of NP (Bouhassira, 2019; Mulvey et al., 2014). However, this assessment is complicated by individual differences in sensitivity (Coghill & Eisenach, 2003) and the lack of reliability in these measures that often include the evaluation of the impact of NP on activities of daily living and quality of life (S. M. Smith et al., 2016). This highlights the critical need for objective data to assess pain and support the management of pain perception. The identification of a biomarker(s) that could complement patient reporting and serve as a correlate to the neurobiological processes underlying painful conditions would be an important tool in identifying effective treatments. This could also support the aim of reliably diagnosing NP. Furthermore, biomarkers that are directly related to the presence and severity of NP could lead to (a) successful mechanism‐based treatment approaches to alleviate the need for long‐term use of opioids, (b) significant reduction in the healthcare costs worldwide, and (c) improvements in the quality of life of NP patients.

The FDA (FDA‐NIH Biomarker Working Group, 2016) describes a biomarker as a “defined characteristic that is measured as an indicator of normal biological processes, pathogenic processes, or responses to an exposure or intervention, including therapeutic interventions.” Biomarkers that have been studied in NP include plasma and cerebrospinal fluid biomarkers (lipid mediators, nerve growth factor, BDNF, tumor necrosis factor alpha, interleukins, and neurotransmitters like gamma‐aminobutyric acid and glutamate) (Bönhof et al., 2018; Gunn et al., 2020), skin biopsy (Bönhof et al., 2018; Sisignano et al., 2019; S. M. Smith et al., 2017), genetic biomarkers (point mutations in the gene encoding of TRPV1 and TRPA1, SCN10A and SCN11A), (Bönhof et al., 2018; Sisignano et al., 2019), sensory biomarkers (quantitative sensory testing) (Sisignano et al., 2019; S. M. Smith et al., 2017), and imaging biomarkers (resting‐state brain activity, evoked activity with ongoing clinical pain) (Miesen et al., 2019; Sisignano et al., 2019; S. M. Smith et al., 2017).

For approximately two decades, BDNF has attracted attention as a potential biomarker for NP because it promotes neuronal growth, maintenance, survival, and neurogenesis (Barde et al., 1982; Binder & Scharfman, 2004; Leibrock et al., 1989; Park & Poo, 2012; H. Zhao et al., 2017). BDNF is a member of the neurotrophic factor family (H. Zhao et al., 2017), has been identified as an important pain modulator (Merighi et al., 2008; Pezet et al., 2002; Vanelderen et al., 2010) and it regulates central and peripheral synaptic plasticity (Binder & Scharfman, 2004; Panja & Bramham, 2014; Park & Poo, 2012; H. Zhao et al., 2017). BDNF synthesis is initiated from pre‐pro‐BDNF, which is cleaved to mature BDNF, and is secreted both by presynaptic and postsynaptic terminals with its secretion dependent on neuronal activity (Autry & Monteggia, 2012; Binder & Scharfman, 2004; Park & Poo, 2012). It has also been implicated in neuropathic (Ding et al., 2015; Pezet & McMahon, 2006; Pezet et al., 2002; Tateiwa et al., 2018; Wu et al., 2021; H. Zhang et al., 2017; X. Zhang et al., 2011) and inflammatory pain mechanisms (Ishikawa et al., 2015; Sikandar et al., 2018; J. Zhao et al., 2006) because of its important role in sensory neurotransmission in spinal and supraspinal level nociceptive pathways.

It is plausible that BDNF initiates compensatory processes that facilitate recovery or alleviate the adverse chronic effects of injury or disease to the central and peripheral nervous system. Furthermore, BDNF can act as a pain mediator and modulator at different sites in the central nervous system including dorsal root ganglion, spinal cord, and supra‐spinal sites. Lastly, because of its involvement at the dorsal horn level, previous studies have also implicated its role in central sensitization (Alles et al., 2021; Biggs et al., 2010; Sikandar et al., 2018; Vanelderen et al., 2010). Furthermore, long‐term BDNF exposure increases the excitability of the dorsal horn and mediates central sensitization of the dorsal horn, which initiates changes in synaptic functioning that may be responsible for the generation of NP (Dai & Ma, 2014; Kerr et al., 1999; P. A. Smith, 2014).

Although BDNF has been proposed as a candidate biomarker of chronic pain, especially NP, there remains a significant gap in our understanding of the physiological mechanisms that lead to changes in BDNF levels measured peripherally. This is partially due to the difficulty in assessing central nervous system BDNF level. In addition, an understanding of this multifactorial experience could lead to the more effective use of personalized medicine approaches to pain management. The purpose of this review is to (a) summarize current findings from human studies that have utilized BDNF as a potential biomarker, (b) briefly outline the role of BDNF in NP by summarizing results from animal studies, and (c) provide a critique of the existing measurement techniques used to assess BDNF with the intention of presenting a case for the most reliable and valid techniques.

As an initial step, Table 1 provides the study characteristics and findings for articles that have investigated group differences at baseline (Table 1a) and group differences across time following an intervention (Table 1b) utilizing BDNF as a biomarker in healthy volunteers, patients with chronic pain, and patients with neuropsychiatric disorders.

**TABLE 1a:** Study characteristics and findings for studies examining baseline group differences in brain derived neurotrophic factor (BDNF) in healthy volunteers, patients with chronic pain and patients with neuropsychiatric disorders

**Author(s)**	**Study population**	**Source of BDNF measurement**	**Mean ± SD BDNF values in pg/ml or ng/ml (as noted)**	***P*‐values Sig or Not Sig**
Stefani et al., 2019	Fibromyalgia (*n* = 117), Osteoarthritis (*n* = 88), Endometriosis (*n* = 36), Chronic tensional type headache (*n* = 33) and Healthy controls (*n* = 41)	Serum	^a^Osteoarthritis: 24.85, Endometriosis: 23.71 Fibromyalgia: 38.60, Chronic tensional type headache: 37.22 and Healthy controls: 22.85 (values in pg/ml)	Sig
Jasim et al., 2020	Chronic temporomandibular disorders myalgia (TMD) (*n* = 39) and Healthy controls (*n* = 39)	Salivary Plasma	TMD group: 3.57 ± 1.47 and Healthy controls 4.62 ± 2.51 TMD group: 263.33 ± 245.13 and Healthy controls 151.81 (values in pg/ml)	Sig Sig
Rocha et al., 2017	Ovarian endometrioma (*n* = 11), other benign ovarian tumors (*n* = 11), deep endometriosis (*n* = 9) and uterine fibroids (*n* = 4)	Plasma	Ovarian endometrioma: 1063 ± 157, other benign ovarian tumors: 537 ± 131, deep endometriosis: 584 ± 138, and uterine fibroids: 216 ± 129 (values in pg/ml)	Sig
Pillai et al., 2010	Male patients with Schizophrenia (*n* = 15), Female patients with Schizophrenia (*n* = 19), Male Healthy controls (*n* = 13) and Female Healthy controls (*n* = 23)	Plasma CSF	^b^Lower in patients with Schizophrenia than controls ^b^Lower in patients with Schizophrenia than controls	Sig Sig
Baumeister et al., 2019	Fibromyalgia (*n* = 89), Healthy controls (*n* = 36)	Plasma	^b^No differences between the two groups	Not Sig
Lang et al., 2004	Male (*n* = 64) and female (*n* = 54) healthy volunteers	Serum	Males: 16.1±7.2 and Females: 16.5±7.4 (values in ng/ml)	Not Sig
Haas et al., 2010	Fibromyalgia (*n* = 30), Healthy controls (*n* = 30)	Plasma	Fibromyalgia: 167.1 ± 171.2 Healthy controls: 113.8 ± 149.6 (values in pg/ml)	Sig
Caumo et al., 2016	Fibromyalgia (*n* = 19), osteoarthritis (*n* = 27), myofascial pain syndrome (*n* = 54) and healthy controls (*n* = 14)		Fibromyalgia: 50.78±16.06, osteoarthritis: 17.91±7.27, myofascial pain syndrome: 29.28±20.01 and healthy controls: 19.00±8.79 (values in ng/ml)	Sig
Deitos et al., 2015	Central sensitivity syndrome absent of structural pathology (*n* = 81), Central sensitivity syndrome with persistent nociception (*n* = 59) and healthy controls (*n* = 37)	Serum	Central sensitivity syndrome absent of structural pathology: 49.87±31.86, Central sensitivity syndrome with persistent nociception 20.44±8.30 and healthy controls:14.09±11.80 (values in ng/ml)	Not reported
Gasparin et al., 2020	Male (*n* = 32) and female (*n* = 24) healthy volunteers	Serum	Males 33.06 ± 11.87 and females 23.71 ± 13.71 (values in ng/ml)	Sig

*Note*: Sig, Significant difference between the two groups with *P* < .05; Not Sig, No significant difference between the two groups.

^a^ No SD values reported;

^b^ BDNF values not reported.

[Correction added on 22^nd^ February 2023, after first online publication: Table 1 and 2 has updated.]

**TABLE 1b:** Study characteristics and findings for studies examining group differences in brain derived neurotrophic factor (BDNF) across time in healthy volunteers, patients with chronic pain and patients with neuropsychiatric disorders

**Author**	**Study population**	**Source of BDNF measurement**	**Mean ± SD BDNF values in pg/ml or ng/ml (as noted)**	***P*‐values Sig or Not Sig**
Gomes et al., 2014	Knee osteoarthritis	Plasma	Before exercise: 7.69 ± 4.45 Immediately following exercise: 12.24 ± 3.80 (values in pg/ml)	Sig
Hanoglu et al., 2021	Alzheimer's disease (N=15)	Serum	Pre rTMS: 372.01 ± 42.41 Post rTMS: 508.61 ± 47.55 (values in pg/ml)	Sig
Cho et al., 2012	Male Healthy volunteers (N=18)	Plasma Serum Platelet	At rest: 3376.87 ± 319.45, ^a^Increase post exercise At rest: 22944.54 ± 9116.57, ^a^Increase post exercise At rest: 77.32 ± 33.89, ^a^Increase post exercise (values in pg/ml)	Sig Sig Sig
Naegelin et al., 2018	Male (*n* = 81) and Female (*n* = 178) Healthy volunteers	Serum	At baseline, Males: 32.34 ± 7.82, Females: 32.85 ± 8.57. At 12 months, Males: 32.95 ± 8.19, Females: 32.98 ± 8.47 (values in ng/ml)	Not Sig Not Sig
Gaede et al., 2014	Healthy volunteers (*n* = 39)	Serum	Pre: 8.2 ± 2.7 Post: 5.6 ± 3.2 (values in ng/ml)	Sig
X. Zhao et al., 2019	Patients with refractory depression receiving rTMS (*n* = 29), Patients acting as controls not receiving rTMS (*n* = 29), Healthy volunteers (*n* = 30)	Serum	Patients receiving rTMS: 4.24±1.12, ^a^Increase post rTMS Patients acting as controls: 4.31±1.14, ^a^Increase post rTMS Healthy volunteers: 16.77±1.07 (values in pg/ml)	Sig Sig
Lang et al., 2008	Male (*n* = 19) and female (*n* = 23) healthy volunteers	Serum	Males: Pre rTMS: 10.05 ± 2.6 vs Post rTMS 10.01 ± 3.68 Females: Pre rTMS: 11.25±4.27 vs Post rTMS 11.38±4.16 (values in ng/ml)	Not Sig
Slusher et al., 2018	Male Healthy volunteers (N=13)	Serum Plasma	^a^Increase post exercise ^a^Increase post exercise (values in pg/ml)	Sig Not Sig

*Note*: SD, standard deviation; rTMS, repetitive transcranial magnetic stimulation; Sig, significant difference between the two groups with *p* < 0.05; Not Sig, No significant difference between the two groups; ^a^BDNF values not reported.

It is evident from the information in Table 1a, 1b that BDNF is typically measured either in serum or in plasma. In addition, studies quantify concentrations of BDNF in pg/ml or ng/ml which makes standardization of the BDNF levels difficult, and these studies present inconsistent findings with a number of studies demonstrating differences at rest between various groups and across time in healthy volunteers, gender, patients with chronic pain, and patients with neuropsychiatric disorders, whereas other studies have not demonstrated these differences. The perplexing nature of these data demand a more intricate examination beginning with the evidence supporting BDNF as a possible mechanistic biomarker for pain perception and then a more sophisticated review of the measurement techniques utilized.

## 2 BDNF RELATED MECHANISM OF ACTION IN NP

**FIGURE 1:**
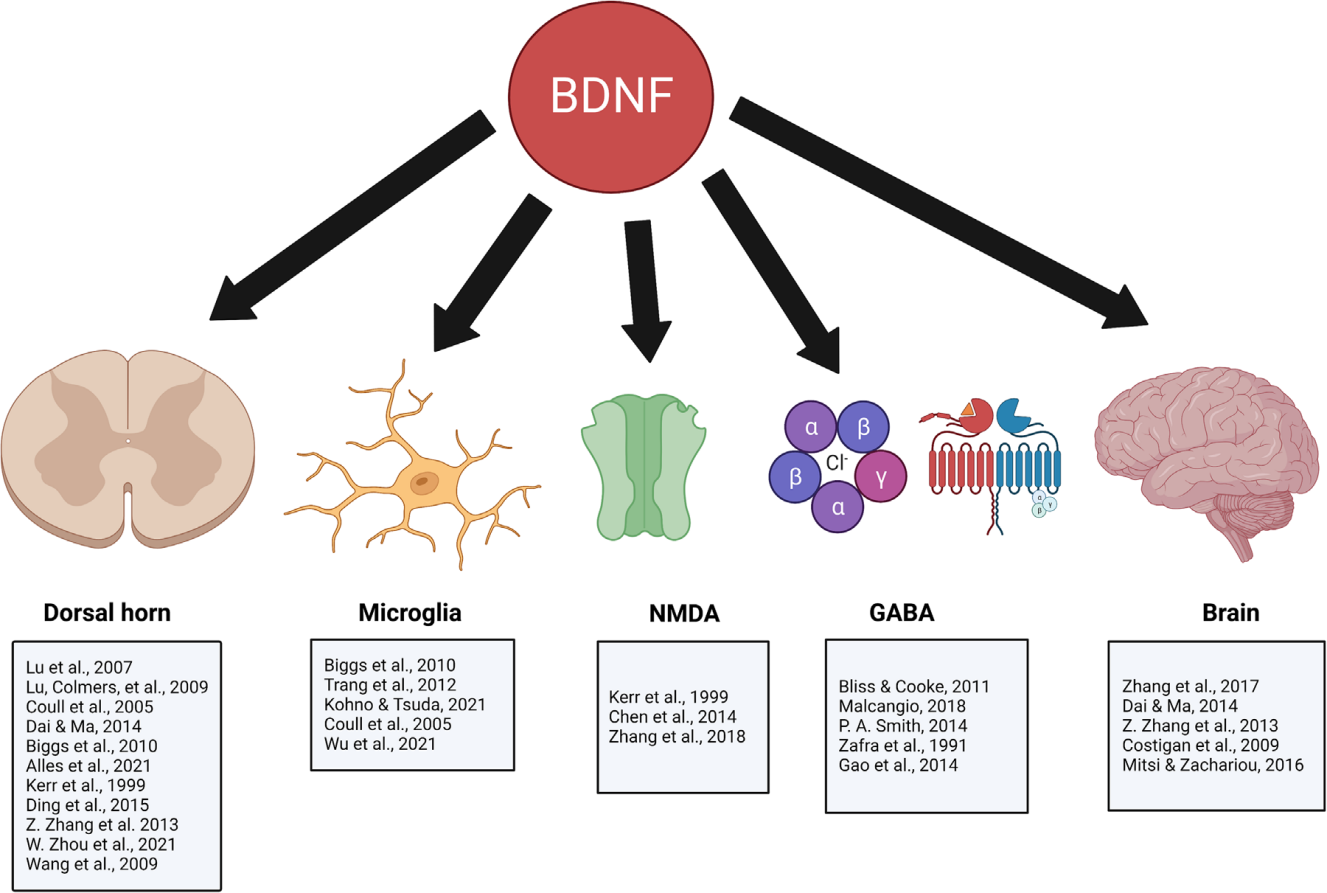
List of studies that support the role of brain derived neurotrophic factor (BDNF) in neuropathic pain with the associated site for the mechanism of action. Created with BioRender.com

BDNF acts as a pain mediator (factor that contributes to the initiation and development of pain) and modulator (factor that regulates pain) and performs its biological functions through two receptors: p75 neurotrophin (pan‐selective p75 neurotrophin receptor) and the TrkB receptor (tropomyosin receptor kinase B or tyrosine receptor kinase B) (Andero et al., 2014; Binder & Scharfman, 2004; Chao & Hempstead, 1995). BDNF also binds with TrkC at a lower affinity, which is primarily activated by neurotrophin 3 (NT‐3) (Ateaque et al., 2022; Mcmahon et al., 1994; Reichardt, 2006). BDNF is released in response to peripheral inflammation and is known as a nociceptive modulator for both pain perception and sensitization at both spinal and supraspinal levels (Merighi et al., 2008; Pezet & McMahon, 2006). p75 is a low affinity receptor while the tropomyosin receptor kinase B (TrkB) receptor is a high affinity receptor (Binder & Scharfman, 2004), and is upregulated in chronic pain states (Pezet et al., 2002, Pezet & McMahon, 2006; Merighi 2008; Smith, 2014; Thibault et al., 2014; Wang et al., 2009). Spinal BDNF‐TrkB signaling has been implicated in studies that have investigated pathological mechanisms for NP (Cao et al., 2020; Coull et al., 2005; Ohira & Hayashi, 2009; Soril et al., 2008; Thibault et al., 2014; X. Wang et al., 2009). This BDNF‐TrkB signaling can modulate neurotransmission and enhance synaptic efficacy both via presynaptic and postsynaptic mechanisms (Binder & Scharfman, 2004; Cheng et al., 2017; Pezet & McMahon, 2006; Yoshii & Constantine‐Paton, 2010). Furthermore, the pronociceptive role of BDNF–TrkB is responsible for the persistent increase in excitability of second order neurons in the spinal dorsal horn contributing to allodynia, hyperalgesia, spontaneous pain, and causalgia that characterize NP and central sensitization (Biggs et al., 2010; Sandkühler, 2009; Woolf, 2007; H. Zhao et al., 2017). Because the focus of this article is on NP (pain induced by injury to the nervous system) and the associated role of BDNF in promoting neuronal growth, survival, and neurogenesis in the nervous system, animal studies that describe the prevalent role of BDNF in the initiation and maintenance of NP at the spinal, peripheral and central levels will be discussed. Figure 1 provides a depiction of the role of BDNF in NP and the site of involvement for its mechanism of action with citations of the supporting literature.

### 2.1 Spinal dorsal horn, dorsal root ganglia and microglia mediated action

Previous studies utilizing NP models have demonstrated that elevated BDNF levels in the spinal dorsal horn contributes to hyperalgesia and central sensitization (Alles et al., 2021; Dai & Ma, 2014; Z. Zhang et al., 2013). Evidence from preclinical studies that utilize peripheral nerve injury models have also revealed that BDNF is synthesized by dorsal horn neurons and causes hyperexcitation of dorsal horn neurons, which results in pain hypersensitivity (Coull et al., 2005; Ding et al., 2015; Sikandar et al., 2018; L. J. Zhou et al., 2011; W. Zhou et al., 2021), an important contributor to NP. Lu et al. (2007 and 2009) describe the role of BDNF in NP using chronic constriction injury models in the rat dorsal horn to illustrate the increased excitability in the dorsal horn. These investigators demonstrated that the excitatory and inhibitory neurons in the substantia gelatinosa of the dorsal horn exhibited altered behavior due to changes in synaptic drive mediated by the release of BDNF with increased excitatory synaptic drive to excitatory neurons and a decrease in the synaptic drive to the inhibitory interneurons. It is critical to consider that central sensitization is an activity‐dependent increase in excitability of dorsal horn neurons (Latremoliere & Woolf, 2009; Woolf & Thompson, 1991), and BDNF expression facilitates this process by promoting a slowly developing increase in excitability and synaptic activity in the dorsal horn. From here, TrkB receptors are activated on second order neurons or primary afferent endings which in turn activate spinal reflexes and primary afferents (Dai & Ma, 2014; Kerr et al., 1999; Lu et al., 2007; X. Wang et al., 2009) causing allodynia, hyperalgesia, and spontaneous pain, defining characteristics of NP. With activation of TrkB receptors, there is a downstream activation of different signaling pathways. For example, nuclear factor kappaB (NF‐ κB) and the mitogen‐activated protein kinases (MAPK), that include the p38, Jun N‐terminal kinase (JNK), and extracellular signal‐regulated protein kinase (ERK) signaling pathways are activated (Cappoli et al., 2020; Liu et al., 2007; Obata & Noguchi, 2004; Pezet & McMahon, 2006; Phạm et al., 2022; Santana‐Martínez et al., 2018). Obata and Noguchi (2004) have also demonstrated that MAPK signaling pathways, specifically ERK and p38 are involved in heat hyperalgesia and nerve injury induced neuroinflammation and neuropathic pain.

Microglia are the resident immune cells in the central nervous system, and their activation following peripheral nerve injury upregulates the purinergic receptors, especially P2 × 4R, causing the phosphorylation of p38‐MAPK that results in the release of BDNF (Ji & Suter, 2007; Trang et al., 2011a; T.T. Zhou et al., 2014) Zhou, 2014; Suter, 2007; Trang, 2011). This facilitates the excitability of dorsal horn neurons and persistence of hypersensitivity contributing to NP (Biggs et al., 2010; Coull et al., 2005; Kohno & Tsuda, 2021; Trang et al., 2011). In the microglia, BDNF can also activate PI3K and ERK kinase pathways that are fundamental for the development of neuropathic pain (Trang et al., 2011). The p38 pathway in the microglia also activates NF‐ κB leading to release of inflammatory mediators like interleukin‐1beta (IL‐1β) and interleukin‐6 (IL‐6) leading to pain hypersensitivity after nerve injury (Ji & Suter, 2007; Obata & Noguchi, 2006). Tumor necrosis factor, another inflammatory mediator involved in NP inhibits long‐term potentiation via NF‐ κB and p38, MAPK and JNK signaling pathways (Butler et al., 2004; Liu et al., 2007). Additionally, activation of the above signaling pathways also causes secretion of reactive oxygen species(X. Gao et al., 2007; I. Lee et al., 2007), and previous studies have highlighted the role of nitric oxide in central sensitization and neuroinflammation (Cury et al., 2011; Schwartz et al., 2008; Teixeira‐Santos et al., 2020)

Elevated levels of BDNF at the level of the dorsal root ganglion (DRG) facilitates pain transmission and contributes to pain hypersensitivity and central sensitization (Wu et al., 2021). Both in NP and inflammatory pain models, increased levels of BDNF in the DRG neurons are correlated with increase in the BDNF levels in the spinal dorsal horn (Pezet & McMahon, 2006; Wu et al., 2021). In the DRG, BDNF/TrkC is primarily expressed in large sensory afferents specifically mechanoreceptors and proprioceptors (Mcmahon et al., 1994). Following nerve injury, Michael et al. demonstrated an increase in BDNF expression in the TrkC and TrkB receptors (Michael, 1999). Similar to the role of microglia in NP, nerve injury also leads to activation and recruitment of macrophages in the DRG (Malcangio, 2019; Tu et al., 2022; Yu et al., 2020; H. Zhang et al., 2016). These macrophages release BDNF and BDNF‐ TrkB plays an important role in both the initiation and maintenance of the mechanical hypersensitivity of NP (Tu et al., 2022; Yu et al., 2020). Yu et al. (2020) also demonstrated that depletion of DRG macrophages prevented the upregulation of BDNF within 24 h of nerve injury in mice (Yu et al., 2020).

Therefore, at the spinal level, BDNF is expressed in microglia, in the neurons, and in nociceptors of the DRG and in the dorsal horn neurons. This BDNF release is maladaptive in that it contributes to central sensitization and NP.

### 2.2 NMDA–Glutamate–GABA receptor–mediated action

BDNF also exerts its effects via interactions with other receptors, neurotransmitters, and ion channels. Specifically, presynaptic BDNF signaling promotes neurotransmitter release and postsynaptically it is involved in enhancing various ion channel function. BDNF modulates synaptic plasticity in an activity dependent manner contributing to long term potentiation (C. S. Wang et al., 2022) via TrkB and downstream signaling cascades, mainly phosphatidylinositol 3‐kinase/protein kinase B, MNK, and the mechanistic target of rapamycin (mTOR)‐signaling (Cappoli et al., 2020; Nijs et al., 2015; Pezet & McMahon, 2006). The presynaptic actions of BDNF can involve synapsins and calcium channels (Cheng et al., 2017; Jovanovic et al., 2000). The BDNF‐ TrkB‐ MNK signaling leads to augmentation of the synapsin 1 phosphorylation causing sustained release of glutamate (Cheng et al., 2017; Jovanovic et al., 2000). Synapsins are proteins that are linked to synaptic vesicles encoding three mammalian genes which generate multiple isoforms and mediate the release of neurotransmitters (Cheng et al., 2017). BDNF enhances presynaptic calcium levels due to the activation of the phospholipase C pathway which activates TRPC channels causing release of calcium from intracellular stores (Cheng et al., 2017; Jovanovic et al., 2000). The release of calcium augments the spontaneous release of glutamate. With regards to GABA release, Cheng and colleagues demonstrated that synapsins inhibit GABA release and calcium influx leads to GABA release similar to glutamate release (Cheng et al., 2017).

Previous studies have described the interaction of BDNF‐TrkB signaling with N‐methyl‐d‐aspartate (NMDA) receptors as an underlying mechanism that contributes to central sensitization of spinal neurons (Kerr et al., 1999; Latremoliere & Woolf, 2009; Trang et al., 2011). Animal models of NP have revealed that BDNF‐TrkB signaling promotes the upregulation of NR2B, a subunit of NMDA receptors via activation of the mTOR pathway (L. Zhang et al., 2016). In addition, BDNF plays a major role in modulating the contributions of the glutamatergic and GABAergic mechanisms responsible for long‐term potentiation of the glutamatergic transmission both presynaptically and postsynaptically (Bliss & Cooke, 2011; Merighi et al., 2008; Tyler et al., 2002). BDNF facilitates excitatory transmission at the dorsal horn by attenuating GABAergic inhibitory neurotransmission that causes a disequilibrium in GABA (y‐aminobutyric acid) levels (J. Gao et al., 2014; Lu et al., 2007, 2009; Zafra et al., 1991). This disinhibition is an important contributor to central sensitization and NP (Malcangio, 2018)

In addition, elevated BDNF contributes to decreased expression of KCC2 (Cappoli et al., 2020; Z. Zhang et al., 2013; S. Zhao et al., 2022). KCC2 is a potassium/chloride cotransporter that controls intracellular chloride concentrations in these neurons causing disruption of neuronal chloride homeostasis. This contributes to spinal disinhibition and promotes the development of pain hypersensitivity and mechanical allodynia, which is commonly observed in inflammatory pain and NP models (Cao et al., 2020; Trang et al., 2011; X. Zhang et al., 2011; J. Zhao et al., 2006). The increase in chloride concentrations shifts the chloride equilibrium potential to a less negative value, and this also contributes to GABA disinhibition (Z. Zhang et al., 2013). This BDNF–KCC2–GABA attenuation leads to NP and central sensitization (Chen et al., 2014; Dai & Ma, 2014; P. A. Smith, 2014). Thus, altered BDNF levels in NP, perturb the balance in potentiation between glutamatergic and GABAergic synapses in the central nervous system (CNS) that contributes to an imbalance in excitatory/inhibitory neurotransmission.

### 2.3 Supraspinal involvement

Due to its role in brain signaling and synaptic plasticity, coupled with its involvement in emotional comorbidities like memory, decision making, and depression, cerebral BDNF in brain areas including the hippocampus, prefrontal cortex, and reward centers including the mesocorticolimbic system (Mitsi & Zachariou, 2016; Navratilova et al., 2012; H. Zhang et al., 2017) has been proposed as a marker of nociception in chronic pain. Brainstem areas like the rostroventral medulla and the nucleus raphe magnus involved in descending pain modulation also contribute to the BDNF‐KCC2‐GABA impairment in the development of chronic NP (Costigan et al., 2009; Dai & Ma, 2014). The nucleus raphe magnus activates the descending pain pathways due to BDNF mediated KCC2 downregulation causing GABAergic disinhibition which plays an important role in the process of central sensitization during the development of chronic pain (Dai & Ma, 2014; Z. Zhang et al., 2013).

Figure 1 includes a list of the studies that address the corresponding site of activity for BDNF at the supraspinal, spinal, and receptor level. Therefore, increased levels of BDNF at different locations in the central nervous system including the spinal dorsal horn, the microglia, and the brain, coupled with its involvement at the receptor level and its connections to neurotransmitters like glutamate and GABA, suggests that enhanced BDNF signaling mediates the pathophysiology of chronic NP. Therefore, these BDNF contributions to the processing of pain offer clues to the mechanisms of central sensitization, hyperalgesia, and mechanical allodynia, and support the proposition that BDNF levels may serve as a biomarker for chronic pain.

## 3 MEASUREMENT OF BDNF

BDNF can be quantified in peripheral whole blood, serum, or plasma, and is stored in the platelets (Fujimura et al., 2002). In addition, the brain is potentially a major contributor to circulating blood levels (Rasmussen et al., 2009) since BDNF freely crosses the blood–brain barrier (Pan et al., 1998). Thus, serum and plasma BDNF are highly correlated with central nervous system BDNF (Klein et al., 2011; Lang et al., 2007; Pan et al., 1998; Pillai et al., 2010). For example, in a study on rats, Karege et al. found a positive correlation (*r* = 0.81, *p* < .01) between serum and cortical BDNF concentrations (Karege et al., 2002). Therefore, peripheral blood BDNF levels (serum or plasma) have been used as a proxy for central (brain) BDNF levels. However, several studies have demonstrated discrepant results between plasma and serum BDNF values within the same subjects (see Table 1a and b), while other studies have presented relatively high correlations between serum and plasma BDNF levels (Elfving et al., 2010; Klein et al., 2011; Polacchini et al., 2015; Trajkovska et al., 2007). This highlights the challenge of assessing reliable BDNF concentrations in the periphery. Furthermore, more than 90% of blood BDNF is stored in the platelets (Fujimura et al., 2002) and released from the platelets to serum during the clotting process, explaining in part, the differences in serum and plasma BDNF levels (serum BDNF level is about 100‐ 200‐fold higher than that of plasma) (Brigadski & Leßmann, 2020; Fujimura et al., 2002). Radka et al. also showed that there is a strong correlation between serum serotonin, a marker for platelet activation, and serum concentration of BDNF, thus highlighting the release of BDNF during the clotting process described above (Radka et al., 1996). Previous studies have presented a broad range of correlations between plasma and serum BDNF concentrations ranging from *r* = 0.2 to *r* = 0.70 (Bocchio‐Chiavetto et al., 2010; B. H. Lee & Kim, 2009; Terracciano et al., 2011).

Moreover, circulating BDNF levels measured using conventional enzyme‐linked immunosorbent assay (ELISA) kits, lack of standardization has likely contributed to the poor reproducibility of results. With regards to measuring serum BDNF, Polacchini et al. (2015) analyzed five different assays in healthy adults and found interassay variations of 5%–20%. In addition, there were differences in the form of BDNF that the kits were measuring with some kits selectively recognizing mature BDNF, while the others reacted with both pro‐BDNF and mature BDNF. Furthermore, Naegelin and colleagues (2018) have concluded that “BDNF levels can be reliably measured in human serum, that these levels are quite stable over one year, and that comparisons between two populations may only be meaningful if cohorts of sufficient sizes are assembled (Naegelin et al., 2018).” Interestingly, work done by Chacón‐Fernández, et al. (2016) has demonstrated that differences and changes in serum BDNF levels demonstrated in studies on depression and physical activity likely reflect adaptations in megakaryocytes and platelets (retaining or releasing BDNF) (Chacón‐Fernández et al., 2016). Thus, further examination of these adaptations is warranted. Nonetheless, the ability to reliably assess changes in serum BDNF must standardized procedures for serum preparation and critically reviewed measurement techniques (Polacchini et al., 2015). Lastly, Bus et al. (2011) has demonstrated the ability of platelets to release BDNF and sequester BDNF from blood. This activity may result in differences between serum and plasma BDNF levels. Other considerations that can affect the measurement of BDNF in the plasma and serum include (1) gender (Begliuomini et al., 2007; Lommatzsch et al., 2005), genetics (Cash et al., 2021; Egan et al., 2003; Terracciano et al., 2013) and age (2) the timing of measurement (accounting for diurnal variations) (Begliuomini et al., 2008; Jasim et al., 2020; Pluchino et al., 2009); (3) psychological/psychiatric disorders (Bocchio‐Chiavetto et al., 2010; Leyhe et al., 2008; Polyakova et al., 2015; Ventriglia et al., 2013); (4) physical activity (Cho et al., 2012; Gomes et al., 2014; Slusher et al., 2018); (5) duration of the sample storage period (Bus et al., 2011; Naegelin et al., 2018; Trajkovska et al., 2007; Tsuchimine et al., 2014), and (6) role of platelet activation (Bélanger et al., 2021; Karege et al., 2005). Each of these factors can negatively influence the consistency of results. Table 2 provides a summary of the studies that have examined factors that influence the measurement of serum and plasma BDNF.

**TABLE 2:** Factors affecting measurement of serum and plasma BDNF

**Factor**	**Author**	**Study population**	**Serum BDNF Measurement**	**Plasma BDNF Measurement**
**Gender differences**	Begliuomini et al., 2007	Fertile ovulatory women (*n* = 20), amenorrhoeic women (*n* = 15) and postmenopausal women (*n* = 25)	Not measured	Lower levels in amenorrhoeic and postmenopausal women compared to fertile ovulatory women
	Lommatzsch et al., 2005	140 healthy adults (72 men (*n* = 72), women (*n* = 68)	No gender differences when matched by weight	No gender differences when matched by weight
**Age**	Begliuomini et al., 2007	Fertile ovulatory women (*n* = 20), amenorrhoeic women (*n* = 15) and postmenopausal women (*n* = 25)	Not measured	Decrease with age
	Lommatzsch et al., 2005	140 healthy adults (72 men (*n* = 72), women (*n* = 68)	No difference in the two groups	Decrease with age
**Diurnal Variations**	Begliuomini et al., 2008	Healthy Males (*n* = 34)	Not measured	Elevated BDNF levels in males in the morning with lowest levels at midnight.
	Piccinni et al., 2008	Healthy volunteers, men (*n* = 14) and women (*n* = 14)	No impact of diurnal variation in serum BDNF level in both men and women	Elevated BDNF levels in males at 8am with lowest levels at 10pm.
	Cain et al., 2017	Healthy volunteers, men (*n* = 23) and women (*n* = 16)	Not measured	Significant circadian rhythms in 12/16 women and 12/23 men
	Pluchino et al., 2009	fertile ovulatory women (*n* = 10), women undergoing oral contraceptive therapy (*n* = 10) and post‐menopausal women(*n* = 10)	Not measured	Diurnal variation in BDNF levels and changes with hormonal status.
**Medications**	Polyakova et al., 2015	Systematic review and meta‐analysis	Increase in BDNF levels post treatment with antidepressants	No difference
	Ventriglia et al., 2013	624 subjects (266 Patients with Alzheimer's Disease (*n* = 266), Patients with frontotemporal dementia (*n* = 28), Patients with Lewy body dementia (*n* = 40), Patients with vascular dementia (*n* = 91), Patients with Parkinsons disease (*n* = 30), and controls (*n* = 169)	Increased BDNF levels post treatment with mood stabilizers/antiepileptics and L‐DOPA and decrease in levels post benzodiazepines	Not measured
	Leyhe et al., 2008	Patients with Alzheimer's disease (*n* = 19) and age‐matched healthy controls (*n* = 20)	Post treatment with AChE‐inhibitors there was increase in BDNF levels	Not measured
**Storage conditions**	Amadio et al., 2017	Healthy subjects including males (*n* = 3) and females (*n* = 3)	No plateau in levels after 60 min of clotting at room temperature, but a constant increase for 120 min	Not measured
	Trajkovska et al., 2007b	206 healthy subjects (122 women, 84 men)	Storage at −20°C led to decrease in BDNF levels up to 5 years	Not measured
	Tsuchimine et al., 2014	10 healthy volunteers	No change in BDNF levels over time	Increase in BDNF levels over time and changes with storage temperature

### 3.1 Factors affecting the measurement of serum and plasma BDNF

At present, more than 95% of the studies in the literature that have evaluated factors involved in the measurement of BDNF, analyze either serum BDNF and/or plasma BDNF. Below is a summary of the studies that have examined factors that influence the measurement of serum and plasma BDNF.

#### 3.1.1 Role of gender, age, and genetics

Begliuomini et al. (2007) examined changes in plasma BDNF circulating levels in 60 women (20 fertile ovulatory women, 15 amenorrhoeic women, and 25 postmenopausal women) and discovered that women with regular ovulatory cycles present with higher BDNF levels than amenorrhoeic or postmenopausal women (*p* < 0.001) (Begliuomini et al., 2007). Lommatzsch et al. (2005) also observed in their sample of 68 women, that platelet levels of BDNF were found to be higher in the second half of the menstrual cycle and in the postmenopausal period (Lommatzsch et al., 2005). In the same study, an analysis of weight‐matched groups found that women had significantly lower BDNF levels in platelets than men, but no difference was observed for plasma levels. Both studies also noted that plasma BDNF levels for postmenopausal women decreased significantly with increasing age (number of years following menopause). Similar results were observed for serum BDNF by Bus et al. (2011) who found an age‐related elevation of serum BDNF in premenopausal women and an age‐related decrease in postmenopausal women.

Egan et al. (2003) and Hempstead et al. (2015) have previously described the negative influence of BDNF polymorphisms especially Val66Met polymorphism on the BDNF/TrkB signaling pathways, with reduced TrkB activation causing impaired secretion of BDNF in patients with neuropsychiatric disorders. In a meta‐analysis of 11 studies on healthy individuals that evaluated the relationship between the BDNF Val66Met variant and BDNF levels, Terracciano et al. (2013) concluded that there was no correlation between the BDNF Val66Met variant and serum, plasma, and whole blood BDNF levels.

#### 3.1.2 Influence of diurnal variations and circadian rhythms

In another study by the Begliuomini group (2008), males demonstrated elevated plasma BDNF concentrations in the morning, followed by a substantial decrease throughout the day with lowest values observed at midnight (Begliuomini et al., 2008). Piccini et al. (2008) examined plasma and serum BDNF levels at three different times during the day (0800 h, 1400 h, and 2200 h), and similar to Begliuomini and colleagues, noted significant diurnal variation in plasma BDNF levels in men, with peak values in the morning for men and decreasing levels throughout the day with lowest values at 22:00 h. For women, no significant diurnal variations were observed in plasma BDNF levels. In addition, for serum BDNF, Piccinni and colleagues (2008) observed no changes across the three time points and there were no sex differences. Pluchino et al. (2009) investigated the influence of circadian rhythm and hormonal status on plasma BDNF levels in fertile ovulatory women, women on oral contraceptive therapy, and postmenopausal women. He and colleagues detected significant differences in BDNF levels among the three groups. In fertile women, plasma BDNF levels were significantly higher during the luteal phase compared to the follicular phase, whereas for postmenopausal women, BDNF was significantly lower in the follicular phase (Pluchino et al., 2009). Concerning circadian variations, in all the three groups, plasma BDNF levels decreased during the day. To summarize, in both men and women, plasma BDNF can vary greatly across the day. Thus, when assessing plasma BDNF, it may be beneficial to take multiple samples over a 24‐hour period in consideration of diurnal variations in both men and women, and control for hormonal changes in women. Serum BDNF levels are likely resistant to the impact of diurnal, however, no study has evaluated the influence of circadian rhythms on serum BDNF levels.

#### 3.1.3 Psychological/Psychiatric disorders

Studies have demonstrated stress‐induced alterations in BDNF levels with acute stress causing an increase in serum BDNF levels and chronic stress being associated with reduced serum BDNF levels. (Linz et al., 2019; Meng et al., 2011) In two separate studies in healthy participants, examined serum BDNF levels utilizing an acute psychosocial stress paradigm, the Trier Social Stress Test and the results demonstrated an elevated serum BDNF response compared to baseline and a control group. No studies have examined changes in plasma BDNF.

In a meta‐analysis of 57 studies in human subjects comparing serum and plasma BDNF levels in patients with major depressive disorder, bipolar disorder, and healthy control subjects, Polyakova et al. (2015) reported that at baseline, serum and plasma BDNF levels were reduced in these patients with major depressive disorder and bipolar disorder compared to healthy controls. In the same article, Polyakova and colleague performed a second meta‐analysis that included 553 patients with major depressive disorder who received treatment for 2–8 weeks. They concluded that serum BDNF levels were significantly higher in treatment responders and remitters compared to nonresponders. Only seven studies reported plasma BDNF levels, and no differences were observed in the treatment responders and nonresponders (Polyakova et al., 2015).

In a study looking at changes in serum BDNF levels in patients with Alzheimer's disease before and after 15 months of treatment with acetylcholinesterase inhibitors, Leyhe et al. (2008), reported that serum BDNF levels were higher post treatment. In a subsequent study by Ventriglia et al. (2013), treatment with mood stabilizers/antiepileptics and L‐DOPA, increased serum BDNF levels, whereas patients administered benzodiazepine demonstrated a decrease in serum BDNF. These results highlight the importance of controlling for the use of medications.

#### 3.1.4 Physical activity and exercise training

A majority of the studies evaluating the changes in BDNF levels post exercise have measured serum BDNF. These studies consistently demonstrate an increase in serum BDNF following an acute bout of exercise in healthy individuals. The fact that circulating BDNF is a good surrogate for changes in CNS plasticity and cognition has led some investigators to propose this as a mechanism for explaining the relationship of physical activity and cognitive function. Several studies have demonstrated increases in both serum and plasma BDNF post exercise (Dinoff et al., 2017; Rasmussen et al., 2009; Slusher et al., 2018). In an interesting study, Slusher et al. (2018) investigated the role of plasma and serum BDNF following high intensity interval training on executive function in healthy college aged males, and revealed a significant increase in serum BDNF concentrations post exercise but no difference in plasma BDNF (Slusher et al., 2018). Reycraft et al. (2020) only measured plasma BDNF levels after exercise at different intensities (including moderate‐intensity continuous training at 65%VO_2max_, vigorous‐intensity continuous training at 85%VO_2max_, and sprint interval training) and observed that plasma BDNF levels increased immediately after exercise for all the groups with the greatest increase seen in the sprint interval training group (Reycraft et al., 2020). These increases in plasma BDNF levels were short‐lived with plasma concentrations recovering 30–90 min postexercise for all the groups. In a previous study, Gilder et al. (2014) demonstrated that serum BDNF levels recover more quickly than plasma BDNF levels (30 vs. 90 min) in individuals with high compared with low‐fat free mass post completion of an incremental graded exercise test. This study suggests that the time required for BDNF recovery post exercise is dependent on the biofluid from which the BDNF was quantified, namely serum and plasma and body composition.

#### 3.1.5 Storage conditions

The time from blood sample collection to processing and the temperature at which the sample is stored can influence BDNF levels. During the coagulation process, activation of platelets causes a rapid release of BDNF from platelets into serum within the first hour at room temperature. This suggests that the length of clotting time constitutes a critical methodological issue when measuring the concentration of BDNF, in particular in serum. Therefore, it is important to evaluate preanalysis conditions (e.g., preparation time and temperature) to ensure that BDNF analyses across studies assess similar physiological events. Gejl et al. (2019) noted that BDNF levels measured in serum samples increased significantly with time during the first hour between collection and centrifugation, and subsequently became relatively stable (Gejl et al., 2019). Similar results were seen in a study by Tsuchimine et al. (2014), where BDNF measured in serum increased during the first hour of coagulation at 25°C and were relatively stable with a clotting time between one and 48 h. In contrast, a study by Amadio and colleagues (2017), demonstrated that serum BDNF samples incubated at 37°C, reached a plateau after 30 min, whereas 120 min were necessary to obtain similar BDNF levels at room temperature. Furthermore, Wessels and colleagues (2020) noted that the type of plasma separator tube, storage duration, and number of freeze–thaw cycles can impact the quantification of plasma BDNF concentration. In addition, plasma stored at −80°C compared to −20°C tends to have less variability in mean BDNF concentrations. More specifically, storing plasma BDNF for up to 6 months at either −20 or −80°C was shown to have reproducible results (Wessels et al., 2020). In addition, Trajkovska et al. (2007) demonstrated that serum BDNF levels were stable up to 1 year after being stored at −20°C, but the levels significantly decreased after 5 years of storage and Bus et al. (2011) observed similar decreases after 3.5 years when it was stored at –85°C. Thus, a possible disadvantage of measuring BDNF in serum may be a decline in BDNF levels after long‐term storage of serum, which may not occur for BDNF stored in plasma.

#### 3.1.6 Impact of platelet activation on plasma BDNF levels

A possible confounder in the blood that can impact plasma levels of BDNF is clotting and platelet activation due to the storage of BDNF in platelets. Platelet activation or clotting can release large quantities of BDNF into the bloodstream, which causes the release of platelet factor 4 and the surface expression of P‐selectin. Belanger et al. (2021) demonstrated that higher platelet activity measured using soluble P‐selectin in plasma was associated with significantly higher plasma BDNF concentrations in individuals with and without coronary artery disease. In a study in patients with depression, Karege et al., 2005 investigated whether serum BDNF levels are dependent on platelet reactivity and determined that serum BDNF is independent of platelet reactivity but plasma BDNF levels were accompanied by increase in platelet factor 4, a marker of platelet reactivity. Schneider et al. 1997 examined the role of coagulation factors on platelet activation by evaluating the binding of coagulation factors to the platelet surface and observed that, anticoagulants such as heparin, sodium citrate, and oxalate can influence platelet activation which can influence BDNF release. Another factor that can cause increase BDNF release from platelets is presence of agonists like thrombin, collagen, Ca^2+^ and shear stress (Serra‐Millàs, 2016). It is critical to keep in mind that even with agonist stimulation, only 30–40% of BDNF in platelets is secreted and the other 70% that is present in cytoplasm is never released (Fujimura et al., 2002). Lastly, comorbidities like depression and cardiac abnormalities can influence platelet activation, thus platelet reactivity, assessed by examining platelet factor 4 and or P‐selectin, should be examined in these patients when quantifying BDNF. Galeano et al. (2015) demonstrated that when corrected for hemoconcentration, BDNF levels increased in the whole blood and in the serum 24 hours after exercise compared to baseline, but plasma levels did not significantly change at baseline and at 24 h post exercise. Furthermore, correlation analyses revealed that serum BDNF levels were highly correlated to whole blood levels whereas plasma levels were not.

To summarize, a majority of studies have utilized measurement of serum BDNF as a marker of BDNF levels in healthy controls and in patients with neuropsychiatric conditions, in response to stress, following exercise, and the following the administration of medication. In addition, serum BDNF is more stable and reproducible than plasma BDNF, in particular when considering the impact of diurnal and circadian variations, psychotropic medications, and blood volume changes in response to exercise on plasma BDNF levels. Both serum and plasma levels are sensitive to changes with age, bodyweight, and menstrual cycle phase (hormonal influences in women). It is also important to consider that alterations in serum BDNF have been observed with various clotting times and storage temperatures. Genetic associations linked to the Val66Met polymorphism have not been found to influence the measurement of serum and plasma BDNF.

Below is a list of factors to consider when deciding upon a valid and reliable research protocol.

1. Sex and gender differences (hormonal status for women)
2. Age (older individuals, especially women present with lower BDNF concentrations at baseline)
3. Diurnal variations and circadian rhythms (report time of the day and consider collecting samples at different time points during the day)
4. Assess the use of medication(s)
5. The use of 1 h for clotting time and store samples at −20 to −80°C
6. If measuring in a clinical population with platelet impairment, measure platelet factor 4 and or P‐selectin to evaluate platelet reactivity.

## 4 CONCLUSION

Concerning chronic pain, there is no conclusive evidence supporting the notion that changes in BDNF levels are causative or a consequence of chronic pain conditions, including NP and musculoskeletal pain in humans. In addition, the documented inconsistent results across studies between plasma BDNF and serum BDNF may be attributable to differences in the constitution of plasma and serum. More specifically, BDNF is largely stored in platelets and is released from activated platelets to the serum during the clotting process. This explains the lower concentration of BDNF in plasma compared to serum, and the timing of the changes in serum and plasma BDNF following activities that activate platelets. Based on current evidence, we would recommend assessing serum BDNF levels to quantify peripheral BDNF as they are more stable and sensitive to changes than plasma BDNF. Future studies should clarify serum and plasma responses to various stimuli, and define a standard protocol for the measurement of peripheral BDNF. In addition, large prospective studies are needed to address the methodological confounds and generalizability for utilizing serum BDNF as a biomarker for chronic pain, and specifically for NP diagnosis and response to treatment.

## CONFLICT OF INTEREST STATEMENT

The authors declare no conflict of interest.

### PEER REVIEW

The peer review history for this article is available at https://publons.com/publon/10.1002/brb3.2903.

## Data Availability

Data sharing is not applicable to this article as no new data were created or analyzed in this study.
